# Synchronous diffuse large B-cell lymphoma of the stomach and small cell lung carcinoma: A case report

**DOI:** 10.1097/MD.0000000000008873

**Published:** 2017-12-15

**Authors:** Jia Li, Changli Zhou, Wanqi Liu, Xun Sun, Xiangwei Meng

**Affiliations:** a From the Department of Gastroenterology, First Hospital of Jilin University; b From the Department of Internal medicine, Nursing School of Jilin University; c From the Department of Gastroenterology; d From the Department of Pathology; e From the Department of Gastroenterology, First Hospital of Jilin University, Changchun, Jilin, China.

**Keywords:** large B-cell, lymphoma, non-Hodgkin, stomach neoplasms, synchronous neoplasm, small cell lung carcinoma

## Abstract

**Rationale:**

The synchronous occurrence of lung cancer in patients with gastric neoplasms is relatively uncommon, especially the cases of synchronous coexistence of small cell lung carcinoma and diffuse large B-cell lymphoma of the stomach.

**Patient concerns:**

We encountered a case of synchronous primary small cell lung carcinoma and diffuse large B-cell lymphoma of the stomach. A 63-year-old patient with a 7.5 × 5.09 cm mass in the superior lobe of the right lung diagnosed with small cell lung cancer and synchronous diffuse large B-cell lymphoma of the stomach.

**Diagnoses:**

The diseases were diagnosed by the pathological biopsy and immunohistochemical methods.

**Interventions:**

As the patient received CHOP chemotherapy, pulmonary function deterioraed. Etoposide was added to the chemotherapy.

**Outcomes:**

However, after the first treatment, chest computed tomography showed that the mass in the superior lobe of the right lung had increased to 8.5 × 5.2 cm.

**Lessons:**

This report draws attention to the fact that the treatment of synchronous tumors is a challenge.

## 1 Introduction

Small cell lung cancer (SCLC) is an aggressive disease that accounts for about 13% of lung cancer cases.^[1]^ Although cases of synchronous lung cancer with other organ malignancies are relatively common,^[2–5]^ cases of synchronous SCLC with other carcinomas are rarely reported.^[6]^ Diffuse large B-cell lymphoma (DLBCL) of the stomach has been reported to be observed in 45–50% of all cases of gastric lymphoma.^[7]^ Herein, we report a case of synchronous stage IV SCLC with primary diffuse large B-cell lymphoma of the stomach. For this very rare condition, finding a reasonable treatment therapy was a challenge.

## 2 Case report

**Figure 1:**
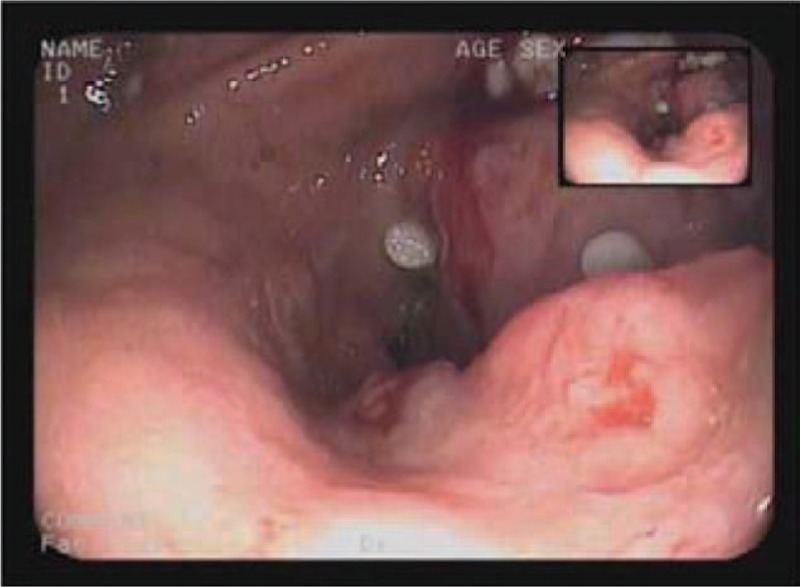
Gastroscopy showed food retention in the stomach, gastric antrum/angle deformation, and that the posterior wall of the antrum had a rough uplift, surface contamination, crisp surface and it bled easily.

A 63-year-old nonsmoking woman was admitted owing to the diagnosis of a mass measuring 7.5 × 5.09 cm in the right main bronchus. She had a history of cough, expectoration, and sputum with blood and chest pain. Chest computed tomography (CT) showed a lung mass in the right upper lobe with right hilar/mediastinal lymph node metastasis and right upper lobe obstruction. Abdominal CT showed the stomach lesions had changed, with increased fat clearance density. The border between the lesion and the inner edge of the left lobe of the liver and pancreatic head boundary was unclear, with multiple swollen peripheral lymph nodes in the lesser curvature of the stomach around the gastric antrum. Gastroscopy showed food retention in the stomach, gastric antrum/angle deformation, and that the posterior wall of the antrum had a rough uplift, surface contamination, and invasion into the pyloric antrum mucosa. The lesion had a rough, crisp surface and it bled easily (Fig. 1). The gastric wall had stiffened with disappearing peristalsis.

**Figure 2:**
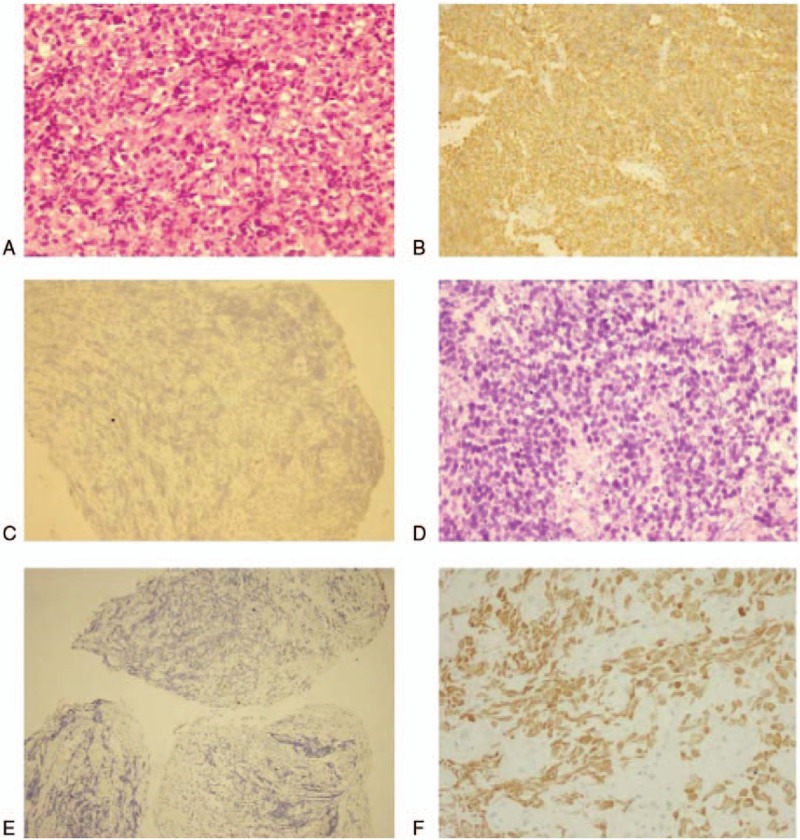
Pathological (A) (10 × 40) examination of the lesion in the antrum. Immunohistochemical results showed CD20(+) (B) (10 × 20), and TTf-(−) (C) (10 × 20). Bronchial biopsy (D) (10 × 40) identified a typical SCLC. Immunohistochemistry of the lung lesion showed CD20(−) (E) (10 × 10) and TTf(+) (F) (10 × 40). SCLC = small cell lung cancer.

Pathological (Fig. 2A) examination of the lesion in the antrum indicated non-Hodgkin's lymphoma, WHO classification of DLBCL. Immunohistochemical results showed Ki-67(+90%), CD20(+) (Fig. 2B), CD79(+), BCL-2(+), BCL-6(+), MUM-1(+), LCA(+), and TTf(−) (Fig. 2C).

**Figure 3:**
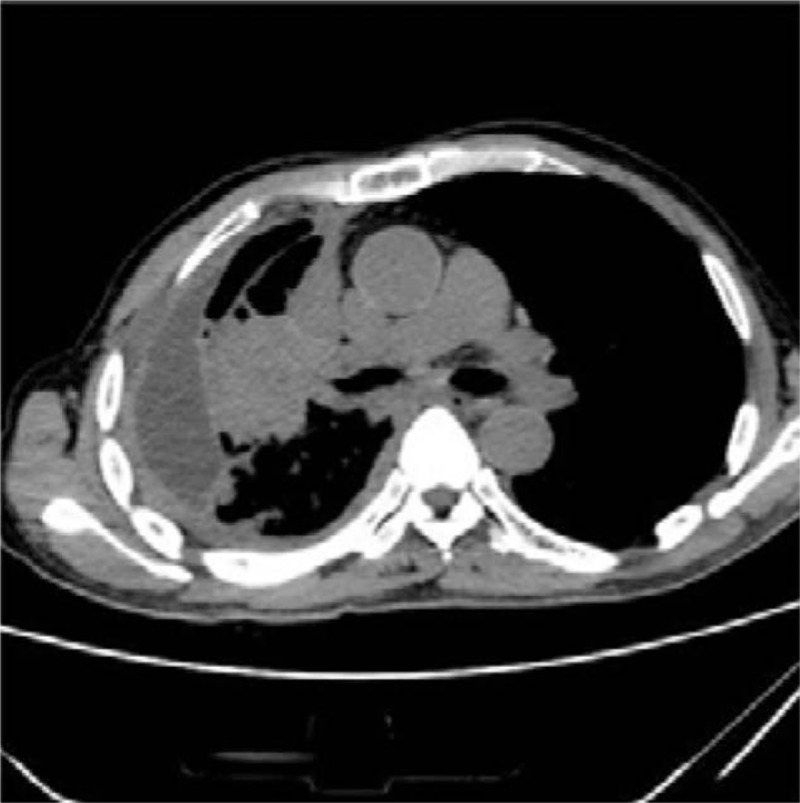
The mass in the right upper lobe, 8.5 cm × 5.2 cm, with inflammation, atelectasis, exacerbating.

The patient received CHOP chemotherapy. On the second day, the patient presented with grade II respiratory failure. Two days later, CHOP chemotherapy was combined with etoposide chemotherapy. After completion of chemotherapy, chest CT identified central lung cancer of the upper lobe, the mass in the right upper lobe had grown to 8.5 cm × 5.2 cm, with inflammation, atelectasis exacerbating (Fig. 3).

**Figure 4:**
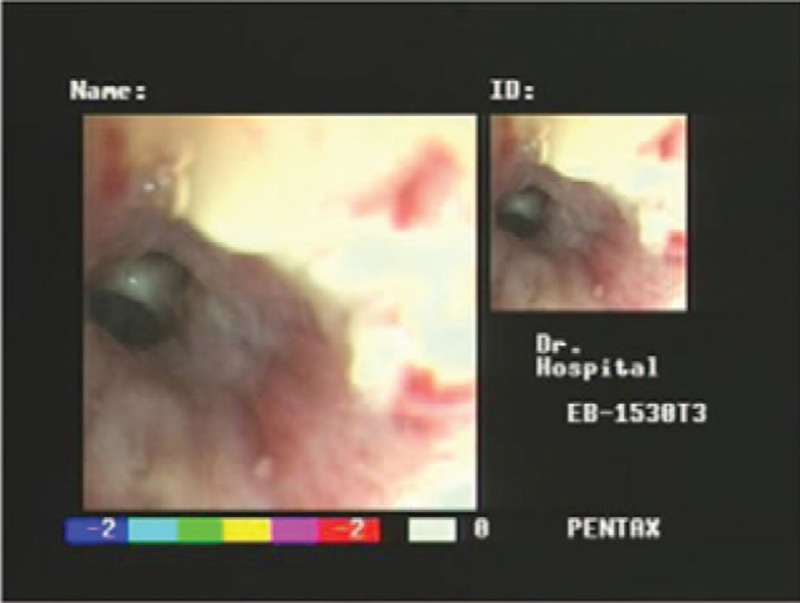
Bronchoscopy showed a rough opening in the right main bronchial mucosa, a cauliflower-like neoplasm that was crisp and bled easily.

During the disease course, the patient still had cough and repeated episodes of hemoptysis. Visible cancer cells were found on sputum cytology. Bronchoscopy (Fig. 4) showed a rough opening in the right main bronchial mucosa, a cauliflower-like neoplasm that was crisp and bled easily, and constriction of the subsections of the right lung's bronchial lumen. Bronchial biopsy (Fig. 2D) identified a typical SCLC. Immunohistochemistry of the lung lesion showed Ki-67(+90%) and CD20(−) (Fig. 2E), and TTf(+) (Fig. 2F). After failure of CHOP combined with etoposide, the patient declined further treatment and was not followed-up after discharge. The patient and her family gave informed consent and agreed to participate in this case report. On the other hand, our case report does not need ethical approval from ethics committee or institutional review board.

## 3 Discussion

Owing to the differences between the pathogenesis of SCLC and DLBCL of the stomach, the treatment is different.^[8,9]^ Although there is no curative modality, combined chemotherapy remains the primary treatment for all stages of SCLC.^[10]^ One patient received targeted receptor tyrosine kinase inhibitors treatment for SCLC and a gastric stromal tumor but died after 11 months.^[2]^ The median survival for patients with limited-stage disease is currently 15 to 20 months, with 20–40% surviving to 2 years, and for those with extensive-stage disease, the values are 8 to 13 months and 5%, respectively.^[11]^ Surgery is traditionally the first-line therapy for the management of gastric DLBCL. However, chemotherapy (CHOP or R-CHOP) alone or chemotherapy followed by radiotherapy produced similar or better results compared with surgical resection.^[12–14]^ More than 80% of patients had a satisfactory response to 6 to 8 cycles of R-CHOP or CHOP.^[15]^ Because the clinical treatment of gastric lymphoma has been established, we administered CHOP to our patient first. However, after the treatment, the lung mass had increased to 8.5 × 5.2 cm and there was inflammation and atelectasis. We supposed that the growth of this mass might be caused by the SCLC which was not treated with systemic chemotherapy. However, the patient and family refused any additional treatment because of the economic burden and the patient was lost to follow-up after discharge.

It is worth thinking about whether prioritizing chemotherapy for the gastric lymphoma was reasonable; whether the increase of the lung mass was related to implementation of gastric lymphoma chemotherapy alone; and whether the patient should have received systemic chemotherapy regimens for both the gastric lymphoma and SCLC. Therefore, for the treatment of synchronous cancer, deciding between prioritizing treatment of one of the cancers or some type of combination therapy is a challenge.
